# Living with Systemic Lupus Erythematosus: A Profile of Young Female Patients

**DOI:** 10.3390/ijerph17041315

**Published:** 2020-02-18

**Authors:** Zelmira Macejova, Andrea Madarasova Geckova, Daniela Husarova, Michaela Zarikova, Zuzana Kotradyova

**Affiliations:** 1 1st Department of Internal Medicine, Faculty of Medicine, Pavol Jozef Safarik University, Trieda SNP 1, 040 11 Kosice, Slovakia; kotradyova.z@gmail.com; 2 Department of Health Psychology and Methodology Research, Medical Faculty, Pavol Jozef Safarik University, Trieda SNP 1, 040 11 Kosice, Slovakia; andrea.geckova@upjs.sk (A.M.G.); daniela.husarova@upjs.sk (D.H.); 3 Olomouc University Social Health Institute, Palacky University in Olomouc, Univerzitni 22, 771 11 Olomouc, Czech Republic; 4 Artromac n.o., Rheumatology Outpatient Clinic, Toryska 1, 040 11 Kosice, Slovakia; m.zarikova@gmail.com

**Keywords:** SLE, disease impact, hospitalization, awareness

## Abstract

The aim is to describe a profile of systemic lupus erythematosus (SLE) patient (socio-demographic data, course of disease, health status, and health care utilization, SLE impact on their life, SLE awareness) and to explore the association of patient’s perspective with clinical indicators. Adult patients diagnosed by SLE were recruited in outpatient clinics (*n* = 76, 88% female, data collected in 2012–2016, Slovakia). The association of patients’ perspective (SLE status, health complaints during remission, SLE impact, hospitalizations) with clinical activity (European Consensus Lupus Activity Measurement Index—ECLAM) and inflammatory marker (erythrocyte sedimentation rate—ESR) was assessed by *t*-test for independent variables and one-way ANOVA. Almost 17.9% of patients reported relapse. During remission, they mostly suffered fatigue and pain. Nearly all patients were on chronic pharmacological treatment. Most of the patients assessed SLE impact on their life as restrictive (56.9%) or very restrictive (23.1%). The most frequent source of information was their physician, and 67.2% reported that they have sufficient information about the disease and its treatment. Only the association of SLE status and hospitalization with clinical activity (ECLAM) and inflammatory marker (ESR) were confirmed. With recent improvements in diagnostics and therapy options, the prognosis for patients with SLE has improved. Nevertheless, the impact of this disease on all areas of a patient’s life is extensive.

## 1. Introduction

Systemic lupus erythematosus (SLE) is defined as a chronic autoimmune disorder that is characterized by a typical multiorgan involvement pattern that is of great significance when it involves vital organs such as the kidney, cardiovascular system, and the central nervous system. SLE is a disease with a development that is difficult to predict. The disease affects young people, especially young women. The estimated prevalence of SLE in Europe is 0.09%, affecting mostly women of working age (mean age at diagnosis, 33 years) [1]. SLE has a profound impact on the quality of life through reduced physical functioning, effects on mental and cognitive health, and work productivity [2]. Fatigue is an important factor influencing patients’ daily life independent of disease activity [3]. SLE affected women of childbearing age are about nine times more frequent than men [4]. National data from Colombia national health care register estimated a prevalence of 91.9/100,000 subjects, being more frequent in women (89% cases). When adjusted, female and male prevalence has a 7.9:1 female:male ratio [5]. The prognosis of SLE has vastly improved in recent years, and this is thanks to improved diagnostic and therapeutic methods. As many as 95% of patients reach 5-year survival, whereas, in the 1950s, this number amounted to 50% only [6]. But the morbidity and mortality are still higher when compared to that of the healthy population. Many studies show that the quality of life in patients with SLE is lower than in general population, independent of variables: measurement instruments (SF36, SF20, SF20+, and the Quality of Life Scale—QOLS), ethnic origin, or the size of the study group [7]. SLE can affect all aspects of a patient’s life. The quality of life in patients with SLE is lower than in the general population and comparable with other chronic diseases [8].

Since SLE is a relatively rare disease, awareness of the disease in the general population is low. Authors Haikel and Tulaihi [9] in their study, pointed out that 56.8% of participants had heard the term SLE and the most common way that they recognized the disease was through the internet. There were no statistically significant differences in awareness about SLE between males and females, but there were statistically significant differences according to education levels [9].

SLE is a disease of young people, so it is assumed that it affects not only the current state of the patient but also his education, the ability to work, and social and family life. The evaluation of the patient’s clinical condition by a physician is different from that of the patient. The European Consensus Lupus Activity Measurement Index (ECLAM) is used to assess clinical status. The erythrocyte sedimentation rate (ESR), although not specific, is a sensitive indicator of inflammation. We were wondering whether we could find a match between clinical indicators and subjective assessment of the patient’s condition. Therefore, our aim is to describe a profile of SLE patient, e.g., socio-demographic data, course of disease, health status, and health care utilization, SLE impact on their life, SLE awareness, and to explore the association of patients’ perspective (SLE status, health complains during remission, SLE impact, hospitalizations) with clinical activity (ECLAM), inflammatory marker (ESR) and positivity of antinuclear antibodies (ANA).

## 2. Materials and Methods

### 2.1. Sample and Procedure

The data collection was carried out in the period between years 2012 and 2016 and patients were recruited from the Rheumatology Outpatient Clinic at 1st and 3rd Departments of Internal Medicine of the Faculty of Medicine at the Pavol Jozef Safarik University and the Louis Pasteur University Hospital, and in the Artromac n.o., Rheumatology Outpatient Clinic.

Inclusion criteria were age over 18 years, diagnosis of SLE confirmed. Exclusion criteria were an inability to fill in the questionnaire (due to dementia or mental retardation, inability to read the Slovak language). We recruited 76 patients with a high predominance of females (*n* = 67). SLE is characterized by predominance in females, and to recruit sufficient number of males would require a different approach. Therefore, we focused our analysis only on females.

Data were obtained by questionnaires filled in by patients during their routine visits to the rheumatology outpatient’s clinic and by extracting medical records. The survey did not refer to any particular treatment. Patients were not enrolled in the trial. They did not take biological therapy such as belimumab or rituximab. Most patients had taken combination therapy with low-dose prednisolone, hydroxychloroquine, or another immunosuppressant. Patients agreed to participate in the cross-sectional study by signing an informed consent prior to the study. They then filled in questionnaires themselves or with a doctor’s assistance. Regarding the latter, we included those recordings closest to the completion of the questionnaires.

The study was approved by the Ethics Committee of the Louis Pasteur University Hospital in Kosice, Slovak republic (2042/87LPS). All data and information used from the documentation, including demographic and clinical ones, were used in accordance with the ethical standards as laid down in the 1964 Declaration of Helsinki and its later amendments or comparable ethical standards.

### 2.2. Measures

Patients were asked about socio-demographic data, course of disease, health status and health care utilization, SLE related impact on daily activities, and SLE awareness.

Socio-demographic data included age, completed education (university, secondary completed with school-leaving examination, apprentice or elementary), living arrangement (living with partner, living with family relatives, living alone), employment status (having job or studying, other), and disablement (without disablement pension, partial disablement pension, full disablement pension). 

With regard to course of disease, patients were asked when they spotted the first symptoms, when they were diagnosed with SLE, and when they were given a disablement pension. Time between first symptoms and setting diagnose was calculated. 

Health status and health care utilization: Patients were asked about SLE status (remission without symptoms, stabilized, with symptoms), how many times they visited physician due to SLE in the past month, how many times they were hospitalized due to SLE in the past year, if they took pharmacological treatment (yes/no). We also asked them how frequently (never, at least once a week, at least once a month, several times per year) and how intensively (none, moderate, significant intensity of symptom) they suffered from the following health complaints during remission: fatigue, pain, decreased physical activity, cosmetic defects, dry mucosa, sleep difficulties, reduced motor skills, memory problems, depression. Those who reported that they suffer from particular symptoms at least once a week, and usually of significant symptom intensity, were reported as cases. Then we divided them by patients who reported none, at least one, and two and more complaints at least once a week, and usually of significant symptom intensity. Moreover, we collected data on the positivity of antinuclear antibodies (ANA), clinical activity by index ECLAM, and inflammatory marker (ESR) from medical records. The European Consensus Lupus Activity Measurement Index (ECLAM) includes the 15 selected variables, weighted (with some adjustments) according to their respective regression coefficients in the multivariate model. As a combination of 15 clinical and laboratory variables, it is one of the predictors of disease activity in SLE [10]. 

We asked patients to evaluate SLE impact on their life generally (very restrictive, restrictive, moderately, or not restrictive) and with regard to particular daily activities in the past week. They were asked how much SLE restricted following daily activities in the past week (without restrictions, minor restrictions, major restrictions, inability to participate): participate on activities at work or at school, household chores, vigorous activities, or doing sport, meetings with friends or relatives, participation in social events, being in sunshine, family life, relationship with partner, sexual life. Considerable restrictions or inability to participate in particular activities were counted as restrictive impact. 

With regard to SLE awareness, we asked patients if they have sufficient information about SLE (yes/no), and about SLE treatment (yes/no), and what is their main source of this information (physician, other patients, journals, relatives, nurse, internet, TV, or radio). Moreover, we asked them if they were familiar with SLE patient organization (yes/no), and if they were a member of an SLE patient organization (yes/no). 

### 2.3. Statistical Analyses

Various continuous and categorical variables were used to describe the profile of SLE patients. With regard to continuous variables, we used mean, standard deviation, minimal and maximal value, median, and with regard to categorical ones, we used number and percentages. The association of patients’ perspective (SLE status, health complaints during remission, SLE impact, hospitalizations) with clinical activity (ECLAM) and inflammatory marker (ESR) and positivity of ANA was assessed by *t*-test for independent variables and one-way ANOVA.

## 3. Results

### 3.1. Socio-Demographic Profile of SLE Patients

Twenty-two point four percent of patients had only completed elementary or apprentice education and nearly 38.8% had completed secondary education, and the same proportion had completed university education. Half of them lived with a partner, 33.3% with family relatives, and 12.1% lived alone. Similarly, half of our patients were employed or studying, while 46.3% were retired, housewife, or unemployed. Only 38.8% of respondents reported not having disability retirement due to SLE, while 32.8% were given partial disability retirement, and 28.4% were given full disability retirement (Table 1).

### 3.2. Course of Disease

**Figure 1:**
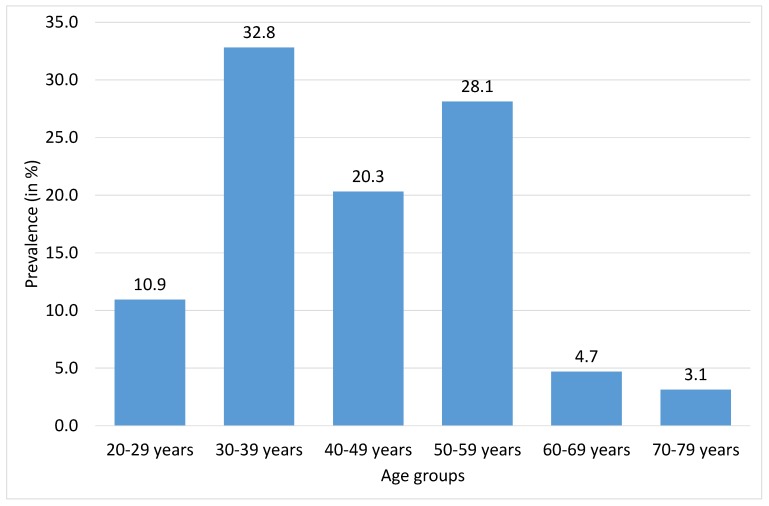
Prevalence of women diagnosed with systemic lupus erythematosus (SLE) by age groups (in %, *N* = 67). missing cases: age groups = 3.

As might be seen in Table 2, most of our patients were of productive age (median = 43.5 years). While there are only 10.9% patients up to 29 years, following age categories between 30 and 59 years comprised more than 81.2% of our sample, and there were only up to 4.7% patients in age group from 60 to 69 years, and only 3.1% of our patients were older than 70 years (see Figure 1).

Moreover, Table 2 showed that symptoms of SLE occurred mostly when patients were 25 years old (median), and most of them were diagnosed when they were 27 years old (median) and they were disabled mostly when they were 31 years old (median). It took an average of a year and a half to set diagnosis when the first symptoms occurred, but mostly it was done within one year (median = 1). The average duration of SLE was 13.1 years (median = 13).

### 3.3. Health Status and Health Care Utilization

Health status and health care utilization by SLE patients is described in Table 3. Most of the patients (64.2%) were stabilized, and 15% were without symptoms, but 17.9% suffer from relapse with symptoms. Nearly all patients were on pharmacological treatment (96.9%). Only 22.7% of patients did not need to visit a physician due to SLE in the past month, but 42.4% visited him/her at least once, and 34.2% visited him/her at least twice in the past month. Around 40.3% of patients were hospitalized due to SLE at least once in the past year. During remission, approximately one-third of patients did not report any health complaints (35.8%), one-third report one complaint (32.8%), and one-third report two and more complaints (31.4%). Most frequently, they report fatigue (35.8%), pain (29.9%), decreased physical activity (25.4%), and cosmetic defects (23.9%). Patients also reported reduced motor skills (14.9%), sleep difficulties (13.4%), or dry mucosa (11.9%). 

### 3.4. SLE Impact

Only 20.0% of patients assessed SLE impact on their life as not restrictive or moderately restrictive, while 56.9% of them found it restrictive and 23.1% very restrictive (see Table 4). Patients reported considerable restrictions or impossibility to participate mainly in vigorous activities (77.3%), activities in sunshine (81.5%), household chores (40.9%), and activities at work or school (32.8%). Most of the patients also reported undesirable side effects of pharmacological treatment (71.6%).

### 3.5. SLE Awareness

**Figure 2:**
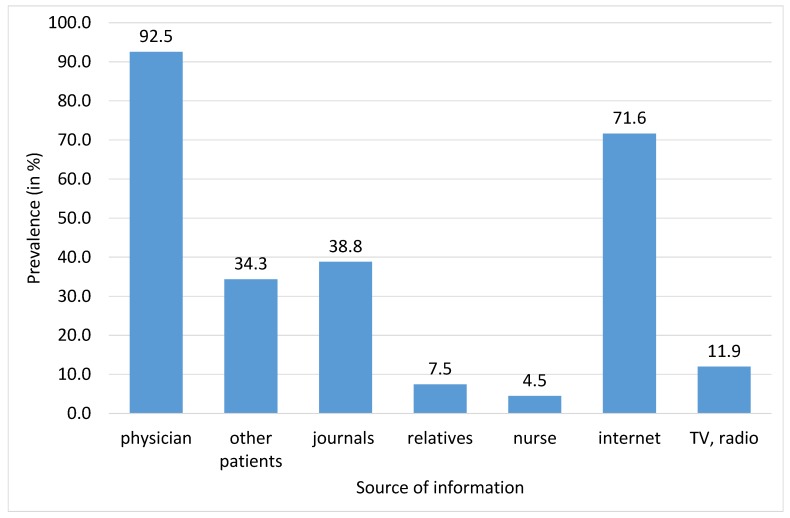
Sources of SLE related information (in %, *N* = 67).

Patients’ awareness about SLE disease and treatment is described in Table 5, and sources of SLE related information are described in Figure 2. Most of the patients reported that they have sufficient information about SLE (67.2%) and SLE treatment (65.7%). The most frequent sources of information were mostly physician (92.5%), internet (71.6%), journals (38.8%), and other patients (34.3%).

### 3.6. The Association of Patient’s Perspective with Clinical Activity

The associations of the patient’s perspective (disablement, SLE status, physician visits, hospitalizations, health complaints, SLE impact) with clinical parameters (ECLAM, ESR, ANA) are described in Table 6. Only the association of SLE status and hospitalization with clinical parameters was confirmed. Patients in remission without symptoms or stabilized patients had significantly lower clinical activity (ECLAM) than patients in relapse with symptoms. Regarding inflammatory marker (ESR) as well as positivity of antinuclear antibodies (ANA), statistical significances were found between all groups of patients. Moreover, patients who did not need to visit a physician in the past month due to SLE had lower inflammatory markers (ESR) than patients who visited him/her at least twice. We did not confirm any significant association between disablement, hospitalizations, health complaints, or SLE impact with inflammatory marker, positivity of ANA, or clinical activity (ECLAM).

## 4. Discussion

This study contributes to the existing body of knowledge on SLE by describing the profile of SLE patients and exploring the association between subjective assessments of the patient’s condition with clinical markers.

The present paper confirmed that SLE is most common in working-age patients, especially in women. This disease has significant adverse effects on many areas in life. The employment rate in patients with SLE is often affected by their disability and frequent sick leaves [11]. Authors Ekblom-Kullberg S. et al. [12] also confirmed that sick leave and absence in work are two or three times more frequent in patients with SLE than in the healthy population. This disease also causes disability, especially in persons with long-lasting SLE [12,13]. A question that still has to be answered is how this disease, disability, and repeated sick leave may affect a potential loss of a job. Author Cristina Drenkard [14] confirmed that almost half of the patients with SLE have lost their jobs over the period of 13 years mainly because of their diagnosis, and only 37% were able to keep their jobs. The literature states that the main negative economic impact was observed especially in persons of younger and middle age [15]. Through the identification of the time from the first symptoms to diagnosis, we tried to find out whether the identification of the diagnosis was delayed, with a consequent delay in the initiation of the efficient therapy. Our survey is in agreement with the literature data [16]. The average time of diagnosis in our patients was 1.5 years. As many as 83% of patients were diagnosed during the second year of their disease, this indicates that the diagnostics of this disease are good and relatively fast despite the fact that the disease may initially cause significant difficulties with regard to differential diagnostics. The literature states that the average time of diagnosis identification after the first symptoms is 2.06 years [16] which is approximately similar to the data we present. 

Moreover, recurrent disease activation, physician office visits, hospitalizations, and sick leave represent a burden for the social and healthcare systems of the country [15]. This results in direct and indirect costs related to disease development, with the indirect costs often being higher [17]. The literature also presents the data confirming that patients with SLE often have suicidal tendencies as compared to the general population [18]. This may be associated with a direct impact of this disease on the Central nervous system (the neuropsychiatric lupus); however, this may also be significantly affected by the patient awareness of the impact of the disease on their common daily and occupational routine activities, inability to find a permanent job, and problems in their families caused by this disease. The areas that are negatively affected by the disease, as reported by the patients, include establishing a family and sexual life. The course of pregnancy in female patients with SLE is difficult to predict. It may become complicated due to the disease as such, or a new SLE attack, as well as due to the therapy. The literature data confirm that pregnancy in patients with SLE represents a risk; nevertheless, pregnancy outcomes of women with low activity prior to their pregnancy significantly improved [19]. 

It is very important that a patient understands the substance of their disease, potential therapies, and factors that may affect the disease course. Cooperation with a doctor and patient awareness are of the key importance in such situations [20]. The awareness among our patients was good. As much as 65.7% of patients stated that they possessed sufficient information on the disease, and 64% of patients stated that they were also aware of the therapy options. Sufficient patient awareness is one of the prerequisites for a successful therapy of SLE [20]. The most frequent sources of information for our patients were their attending doctor and the Internet. Less frequently, they received the information from other patients, from magazines and media (TV), or from other sources. It is surprising that only 4.5% of patients obtained the information form a nurse. Particularly, obtaining patient awareness of their disease through nurses might lead to a reduction in the time burden for doctors. This procedure, however, requires nurses to be educated on this complicated disease, its potential therapies, and prognosis. 

The impact of the disease on the everyday life of patients is significant. Nevertheless, in general, we may state that thanks to an efficient therapy and sufficiently timely diagnostics the prognosis of patients with SLE [21] has lately improved and the impact of the disease on a patient has been mitigated.

SLE is a chronic, progressive disease that leads to irreversible organ changes when activity persists. These changes have an impact on the quality of life, functional status, job title. The current clinical activity of SLE is measured by various indices. One of them is the ECLAM index or measured by humoral indicators (ESR). These indicators reflect the current status. In this way, we explain that we do not confirm any significant association between disablement, hospitalizations, health complaints, or SLE impact with an inflammatory marker (ESR) or clinical activity (ECLAM).

## 5. Conclusions

Systemic lupus erythematosus represents a systemic multi-organ inflammatory disease that especially affects younger people and its consequences significantly affect the everyday lives of patients. Symptoms of SLE occur at a young age and lead to early disability. Nearly all patients were on chronic pharmacological treatment. More than half of the patients visited a physician due to SLE in the past month; 40.3 % of patients were hospitalized due to SLE at least once in the past year. In total, up to 80.0% of patients considered the effect of SLE on their lives to be restrictive or very restrictive. Association of SLE status and hospitalization with clinical parameters were confirmed. By recent improvement in diagnostics and therapy options, the prognosis for patients with SLE has improved and the impact of this disease on their everyday life has been mitigated. Nevertheless, the impact of this disease on all areas of a patient’s life is extensive. 

## Figures and Tables

**Table 1:** Socio-demographic profile of systemic lupus erythematosus (SLE) patients (Slovakia 2012–2016, *n* = 67, female).

	*N* (in %)
Education	
University	26 (38.8)
Secondary (school-leaving examination)	26 (38.8)
Elementary or apprentice	15 (22.4)
Living	
with partner	36 (54.5)
with family relatives	22 (33.3)
alone	8 (12.1)
Having job or studying	
Student or employed	36 (53.7)
Other	31 (46.3)
Disablement due to SLE	
Without	26 (38.8)
Partial disability retirement	22 (32.8)
Full disability retirement	19 (28.4)

missing cases: living with = 1.

**Table 2:** Course of SLE disease (Slovakia 2012–2016, *n* = 67, female).

	Mean (SD); (Min-Max); Median
Age	44.03 (12.48); (23–79); 43.50
Age when first symptoms occurred	28.37 (11.88); (9–74); 25.00
Age when diagnosed SLE	29.92 (11.42); (10–74); 27.00
Age when disability retirement was given	33.05 (10.19); (18–54); 31.00
Time between first symptoms and setting diagnose	1.49 (2.19); (0–12); 1.00
Average duration of SLE	13.1 (8.1); (1–42); 13.00

Missing cases: age = 3, age of first symptoms = 4, age when diagnosed = 4, age of disablement = 3 and *n* = 26 disability retirement was not given.

**Table 3:** Health status and health care utilization (Slovakia 2012–2016, *n* = 67, female).

	*N* (in %)
SLE status	
Without symptoms	10 (14.9)
Stabilized	43 (64.2)
Relapse with symptoms	12 (17.9)
Pharmacological treatment of SLE	
No	2 (3.1)
Yes	63 (96.9)
Visited physician in the past month due to SLE	
None	15 (22.7)
Once in the past month	28 (42.4)
At least twice in the past month	23 (34.2)
Hospitalized in the past year due to SLE	
No	40 (59.7)
Hospitalized at least once in the past year	27 (40.3)
Health complaints during remission (at least once a week, significant symptom intensity)	
Fatigue	24 (35.8)
Pain	20 (29.9)
Decreased physical activity	17 (25.4)
Cosmetic defects	16 (23.9)
Dry mucosa	8 (11.9)
Sleep difficulties	9 (13.4)
Reduced motor skills	10 (14.9)
Memory problems	4 (6.0)
Depression	2 (3.0)
No health complaint	24 (35.8)
One health complaint	22 (32.8)
Two and more health complaints	21 (31.4)
	**Mean (SD); (Min–** **Max); Median**
ANA	2.06 (0.81); (0–3); 2.00
ECLAM	1.94 (2.35); (0–7); 0.00
Inflammation—ESR	36.43 (16.67); (8.00–70.00); 38.00

missing cases: SLE status = 2, pharmacological treatment = 2, visited physician = 1, ANA—antinuclear antibodies; ECLAM—European Consensus Lupus Activity Measurement; ESR—erythrocyte sedimentation rate.

**Table 4:** SLE impact on patients’ lives (in %, *N* = 67).

	*N* (in %)
SLE impact on life generally	
Very restrictive	15 (23.1)
Restrictive	37 (56.9)
Moderately or not restrictive	13 (20.0)
SLE related restrictions in daily activities in the past week (considerable restrictions or impossibility to participate in activity)	
At work or at school	19 (32.8)
Household chores	27 (40.9)
Vigorous activities or doing sport	51 (77.3)
Meetings with friends or relatives	7 (10.6)
Participation on social events	11 (16.7)
Being in sunshine	53 (81.5)
Family life	16 (24.6)
Relationship with partner	13 (20.3)
Sexual life	15 (23.8)
Missing varies from 1 to 9	
Undesirable side effects of pharmacological treatment	
No	13 (19.4)
Yes	48 (71.6)
I do not know	6 (9.0)

missing cases: SLE impact = 2.

**Table 5:** SLE awareness among SLE patients (in %, *N* = 67).

	*N* (in %)
Having sufficient information about SLE	45 (67.2)
Having sufficient information about SLE treatment	44 (65.7)
Be familiar with SLE patient organization	33 (49.3)
Being a member of SLE patient organization	11 (17.5)

missing cases: being a member of SLE patient organization = 4.

**Table 6:** The association of patients’ perspective with inflammatory markers (*t*-test for two independent samples, one-way ANOVA, *n* = 67).

	ECLAM		ESR		ANA	
	Mean (SD)	Significance	Mean (SD)	Significance	Mean (SD)	Significance
**Disablement**		ns		ns		ns
(1) without	1.50 (2.06)		39.39 (15.84)		2.15 (0.88)	
(2) partial disability retirement	2.27 (2.64)		33.27 (17.47)		1.86 (0.77)	
(3) full disability retirement	2.16 (2.39)		35.74 (16.99)		2.16 (0.77)	
**SLE status**		*p* ˂ 0.001		*p* ˂ 0.001		*p* ˂ 0.001
(1) remission without symptoms	0.30 (0.68)	1vs.3, 2vs.3	17.90 (3.07)	1vs.2,1vs.3, 2vs.3	1.30 (0.48)	1vs.2,1vs.3, 2vs.3
(2) stabilized	1.81 (2.05)		37.35 (15.48)		2.07 (0.74)	
(3) with symptoms	4.00 (3.02)		51.17 (11.42)		2.83 (0.39)	
**Visited physician in the past month**		ns		*p* ˂ 0.05		ns
(1) none	1.53 (1.85)		29.60 (13.33)	1vs3	2.07 (0.80)	
(2) once in the past month	1.57 (2.15)		34.61 (15.61)		1.93 (0.72)	
(3) at least twice in past month	2.70 (2.79)		43.65 (17.75)		2.30 (0.82)	
**Being hospitalized in the past year**		ns		ns		ns
(1) no	1.78 (2.06)		34.38 (16.16)		1.93 (0.80)	
(2) at least once in past year	2.19 (2.75)		39.26 (17.29)		2.26 (0.81)	
**Health complaints at least once a week of significant intensity**		ns		ns		ns
(1) none	1.88 (2.36)		34.96 (16.85)		1.88 (0.85)	
(2) one health complaint	2.23 (2.35)		33.18 (14.80)		2.00 (0.82)	
(3) two and more health complaints	1.71 (2.41)		41.24 (17.94)		2.33 (0.73)	
**SLE impact on life**		ns		ns		ns
(1) very restrictive	2.73 (2.82)		39.20 (20.55)		2.33 (0.82)	
(2) restrictive	1.87 (2.28)		37.97 (15.87)		2.00 (0.78)	
(3) moderately or not restrictive	1.46 (2.02)		31.23 (13.31)		2.08 (0.76)	

## References

[1] Ivorra J.A.R., Fernández-Llanio-Comella N., San-Martín-Álvarez A., Vela-Casasempere P., Saurí-Ferrer I., González-de-Julián S., Vivas-Consuelo D. (2019). Health-related quality of life in patients with systemic lupus erythematosus: A Spanish study based on patient reports. Clin. Rheumatol..

[2] Chan K., Dekis A., Clarke A.E. (2012). Hospitalizations in patients with systemic lupus erythematosus: Updated analyses from 2006 to 2011. Arthritis Res. Ther..

[3] Yilmaz-Oner S., Ilhan B., Can M., Alibaz-Oner F., Polat-Korkmaz O., Ozen G., Mumcu G., Kremers H.M., Tuglular S., Direskeneli H. (2017). Fatigue in systemic lupus erythematosus: Association with disease activity, quality of life and psychosocial factors. Rheumatology.

[4] Lisnevskaia L., Murphy G., Isenberg D. (2014). Systemic lupus erythematosus. Lancet.

[5] Fernández-Ávila D.G., Bernal-Macías S., Rincón-Riaño D.N., Gutiérrez Dávila J.M., Rosselli D. (2019). Prevalence of systemic lupus erythematosus in Colombia: Data from the national health registry 2012–2016. Lupus.

[6] Zimmerman-Górska I. (2014). Postępy w Reumatologii Klinicznej.

[7] McElhone K., Abbott J., The L.S. (2006). A review of health related quality of life in systemic lupus erythematosus. Lupus.

[8] Marzena Olesińska M., Saletra A. (2018). Quality of life in systemic lupus erythematosus and its measurement. Reumatologia.

[9] Haikel K.A.B., Al Tulaihi B. (2019). Awareness of systemic lupus erythematosus among primary health care patients in Riyadh, Saudi Arabia. Saudi Med. J..

[10] Vitali C., Bencivelli W., Isenberg D.A., Smolen J.S., Snaith M.L., Sciuto M., Neri R., Bombardieri S. (1992). Disease activity in systemic lupus erythematosus: Report of the Consensus Study Group of the European Workshop for Rheumatology Research. II. Identification of the variables indicative of disease activity and their use in the development of an activity score. The European Consensus Study Group for Disease Activity in SLE. Exp. Rheumatol..

[11] Agarwal N., Kumar V. (2016). Burden of lupus on work: Issues in the employment of individuals with lupus. Work.

[12] Ekblom-Kullberg S., Kautiainen H., Alha P., Leirisalo-Repo M., Julkunen H. (2015). Education, employment, absenteeism, and work disability in women with systemic lupus erythematosus. Scand. J. Rheumatol..

[13] Macejová Ž., Záriková M., Oetterová M. (2013). Systemic lupus erythematosus-disease impact on patients. Cen. Eur. J. Public Health.

[14] Drenkard C., Bao G., Dennis G., Kan H.J., Jhingran P.M., Molta C.T., Lim S.S. (2014). Burden of systemic lupus erythematosus on employment and work productivity: Data from a large cohort in the southeastern United States. Arthritis Care Res..

[15] Utset T.O., Baskaran A., Segal B.M., Trupin L., Ogale S., Herberich E., Kalunian K. (2015). Work disability, lost productivity and associated risk factors in patients diagnosed with systemic lupus erythematosus. Lupus Sci. Med..

[16] Petersen M.P., Möller S., Bygum A., Voss A., Bliddal M. (2018). Epidemiology of cutaneous lupus erythematosus and the associated risk of systemic lupus erythematosus: A nationwide cohort study in Denmark. Lupus.

[17] Kawalec P.P., Malinowski K.P. (2015). The indirect costs of systemic autoimmune diseases, systemic lupus erythematosus, systemic sclerosis and sarcoidosis: A summary of 2012 real-life data from the Social Insurance Institution in Poland. Expert Rev. Pharm. Outcomes Res..

[18] Hajduk A., Nowicka-Sauer K., Smoleńska Ż., Czuszyńska Z., Zdrojewski Z. (2016). Prevalence and correlates of suicidal thoughts in patients with neuropsychiatric lupus. Lupus.

[19] Buyon J.P., Kim M.Y., Guerra M.M., Laskin C.A., Petri M., Lockshin M.D., Sammaritano L., Branch D.W., Porter T.F., Sawitzke A. (2015). Predictors of pregnancy outcomes in patients with lupus: A cohort study. Ann. Intern. Med..

[20] Beusterien K., Bell J.A., Grinspan J., Utset T.O., Kan H., Narayanan S. (2013). Physician-patient interactions and outcomes in systemic lupus erythematosus (SLE): A conceptual model. Lupus.

[21] Ali A., Sayyed Z., Ameer M.A., Arif A.W., Kiran F.N.U., Iftikhar A., Iftikhar W., Ahmad M.Q., Malik M.B., Kumar V. (2018). Systemic lupus erythematosus: An overview of the disease pathology and its management. Cureus.

